# Fidaxomicin for the treatment of *Clostridium difficile* infection (CDI) in at-risk patients with inflammatory bowel disease, fulminant CDI, renal impairment or hepatic impairment: a retrospective study of routine clinical use (ANEMONE)

**DOI:** 10.1007/s10096-018-3344-1

**Published:** 2018-08-11

**Authors:** Maria J. G. T. Vehreschild, Surabhi Taori, Simon D. Goldenberg, Florian Thalhammer, Emilio Bouza, Joop van Oene, Graham Wetherill, Areti Georgopali

**Affiliations:** 1 Department I of Internal Medicine, University Hospital of Cologne and German Centre for Infection Research, Partner Site Bonn-Cologne, Cologne, Germany; 2 King’s College Hospital NHS Foundation Trust, London, UK; 3 King’s College London & Guy’s and St Thomas’ NHS Foundation Trust, London, UK; 4 Department of Infectious Diseases and Tropical Medicine, Division of Internal Medicine I, Medical University of Vienna, Vienna, Austria; 5 Clinical Microbiology and Infectious Diseases, Hospital Gregorio Marañón, Madrid, Spain; 6 Department of Medicine, Ciber de Enfermedades Respiratorias (CIBERES), Complutense University, Madrid, Spain; 7 Astellas Pharma Europe B.V., Leiden, The Netherlands; 8 Astellas Pharma, Inc., Chertsey, UK

**Keywords:** *Clostridium difficile*, Fidaxomicin, Inflammatory bowel disease, Fulminant CDI, Renal impairment, Hepatic impairment

## Abstract

Information is limited or lacking on fidaxomicin treatment of *Clostridium difficile* infection (CDI) in patients with inflammatory bowel disease, fulminant or life-threatening CDI, severe renal impairment, moderate-to-severe hepatic impairment and pregnancy. The ANEMONE study investigated fidaxomicin use in a routine clinical setting, focusing on these medical conditions of specific interest (MCSIs). This retrospective, post-authorisation study reviewed hospital records from Austria, Germany, Spain and the UK (June 2012–June 2015), collecting data from hospital admission to 30 days after last fidaxomicin dose. The primary objective was to identify the proportion of fidaxomicin-treated patients with MCSIs. Secondary objectives were to describe 30-day mortality, changes in ECG and laboratory parameters, fidaxomicin exposure and CDI response (resolution of diarrhoea; 30-day recurrence). 45.3% (261/576) of patients had ≥ 1 MCSI. Thirty-day mortality (post-first dose) was 17.0% (98/576) in the total population and slightly higher (24.6–27.6%) in patients with fulminant CDI or severe renal impairment. 29.6% (24/81) deaths of known cause were attributable to CDI. Of changes in laboratory parameters or ECG findings, only a decrease in leucocyte counts appeared associated with fidaxomicin, consistent with a positive treatment response. Diarrhoea resolved in 78.0% (404/518) of treatment episodes; diarrhoea resolution was lowest in patients with fulminant CDI (investigator-defined, 67.5%, 56/88) and severe renal impairment (68.0%, 68/100). Thirty-day recurrence (18.8%, 79/420) was similar across MCSI subgroups. Although almost half of fidaxomicin-treated patients had ≥ 1 MCSI, the majority of patients in all subgroups had positive responses to treatment, and no particular safety concerns were identified.

**Electronic supplementary material:**

The online version of this article (10.1007/s10096-018-3344-1) contains supplementary material, which is available to authorized users.

## Introduction

*Clostridium difficile* is the leading cause of infectious nosocomial diarrhoea in developed countries [1]. The incidence and severity of *C. difficile* infection (CDI) have increased in recent years [2], alongside increased morbidity, mortality and healthcare costs [3]. The mainstays of CDI treatment over the past 30 years have been metronidazole and vancomycin [4, 5]; more recently, the narrow-spectrum macrocyclic antibiotic fidaxomicin [6–8] has been approved in the USA [9] and the EU [10] for the treatment of CDI.

In two randomised, double-blind, phase III trials, fidaxomicin (one 200-mg tablet orally twice daily for 10 days) demonstrated non-inferiority to vancomycin (125-mg capsules orally four times daily for 10 days) for initial clinical cure of CDI; moreover, fidaxomicin treatment resulted in significantly lower rates of recurrence and higher rates of sustained clinical cure within 30 days of treatment completion [11, 12]. However, patients with CDI and concomitant inflammatory bowel disease (IBD) and patients with fulminant or life-threatening CDI were excluded from these trials [11, 12]. Furthermore, limited data are available on the use of fidaxomicin in patients with CDI who also have severe renal impairment and/or moderate-to-severe hepatic impairment, while data in pregnant women are absent [13]. Hence, there is a lack of evidence on its safety and effectiveness in patients with these conditions. The ANEMONE study aimed to determine the prevalence of these conditions in patient populations treated with fidaxomicin, assess fidaxomicin use in a routine clinical setting and investigate its safety and effectiveness in these specific patient groups.

## Methods

### Study design

**Fig. 1:**
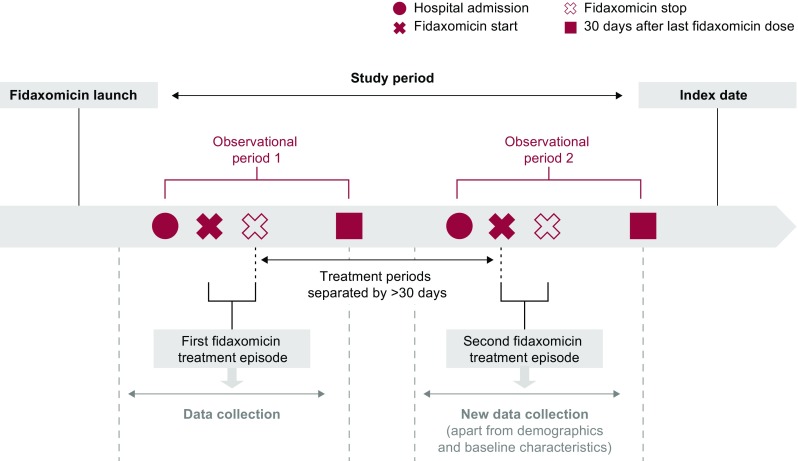
Study overview. The study period was defined separately for each country as the time between the local fidaxomicin launch date and date on which the first site in that country was contacted about this study (the index date). Treatment episode data were collected from the time of CDI-related hospital admission to 30 days after the last dose, or for 40 days after the last prescription date if last dose or other data were missing. Patients could have more than one treatment episode: those > 30 days apart were analysed as separate episodes

This retrospective, multinational, post-authorisation study used anonymised data from hospital records of adult patients who received fidaxomicin during the country-specific eligibility period. The eligibility period lasted from each country’s fidaxomicin launch date to the date the first site in that country was contacted about this study (the index date for all sites in that country). Sites with documented fidaxomicin prescriptions were selected from Austria, Germany, Spain and the UK; across these countries, fidaxomicin launch dates varied from 01 June 2012 to 01 January 2013 and the end of data collection ranged from 10 April 2015 to 18 June 2015. All fidaxomicin-treated patients were enrolled from sites with up to 50 patients; sites with more than 50 eligible patients had 50 selected using a randomisation schedule. Each patient could have more than one episode of fidaxomicin treatment. Treatment episodes were considered distinct if the interval between the last dose of the previous episode and the first dose of the next episode was more than 30 days (Fig. 1).

Data were collected from hospital admission to 30 days after the last dose of fidaxomicin, or, if data were missing, for 40 days after the last prescription date (‘observational period’). Data regarding patient demographics, hospital admission, medical history, prior (≤ 30 days before first dose) and concomitant antibacterial use, and outcomes were collected, if available (Fig. 1).

### Patients

Patients ≥ 18 years old with a fidaxomicin prescription date and corresponding observational period within the country-specific eligibility period were included. Patients were excluded if they had participated in another fidaxomicin study or discontinued fidaxomicin treatment less than 30 days before the index date to ensure that awareness of, or participation in, this study had not influenced clinical practice.

Patients with IBD, fulminant or life-threatening CDI, severe renal impairment, moderate-to-severe hepatic impairment and/or who were pregnant were categorised as having a medical condition of specific interest (MCSI) (Table 1). Patients could present with more than one MCSI.

**Table 1:** Definitions of MCSI

MCSI	Definition
Inflammatory bowel disease	Either Crohn’s disease or ulcerative colitis as noted in the patient records
Fulminant or life-threatening CDI	Assessed both by the principal investigator (fulminant CDI-PI) and, separately, by applying a scoring system (fulminant CDI-SS; see Supplementary Table 2). Fulminant CDI-PI includes pseudomembranous colitis
Moderate-to-severe hepatic impairment	Moderate: baseline serum total bilirubin 34–50 μmol/L; severe: baseline serum total bilirubin > 50 μmol/L
Severe renal impairment	Creatinine clearance ≤ 30 mL/min
Pregnancy	Positive human chorionic gonadotropin test, or positive ultrasound or pregnancy status recorded in the medical notes

*CDI Clostridium difficile* infection, *MCSI* medical condition of specific interest, *PI* principal investigator, *SS* scoring system

### Characterisation of CDI

Severe CDI was defined from records by the principal investigator (PI) or according to European Society of Clinical Microbiology and Infectious Diseases 2009 or 2014 criteria [14]. Fulminant or life-threatening CDI (including pseudomembranous colitis) was primarily identified by the PI (fulminant CDI-PI); a separate scoring system was also used in parallel (fulminant CDI-SS) (Supplementary Table 1) [15].

### Objectives

The primary objective was to identify the proportion of fidaxomicin-treated patients with an MCSI. Secondary objectives were to describe, in patients with MCSIs and the overall patient population, the number and causes of deaths; changes in ECG and laboratory parameters from admission to the end of the observational period (assessments recorded at admission, before first fidaxomicin dose, at last fidaxomicin dose and at the end of the observational period); fidaxomicin exposure (indication, dose, treatment duration); and treatment response (resolution of diarrhoea, time to resolution of diarrhoea, recurrence of diarrhoea within 30 days after end of fidaxomicin dosing and time to recurrence). Resolution of diarrhoea was determined by the clinician according to published guidelines [16].

### Data analyses

#### Sample size

The planned minimum sample size was 512 patients to give sufficient precision to the confidence intervals. Assuming the expected proportion of patients with a specific MCSI to be 10%, 512 patients would provide a precision of 2.6% for a two-sided 95% confidence interval. Sample size calculations were carried out using nQuery Advisor 7.0.

#### Statistical methods

Continuous variables were summarised using descriptive statistics; categorical variables were summarised using frequency tabulations. Two-sided 95% confidence intervals, using exact methods, were provided for the estimated proportion of each MCSI. All analyses were conducted using SAS® versions 9.3 and 9.4 (SAS Institute, Cary, USA). Survival was presented using a Kaplan-Meier plot.

#### Missing data

Missing data were not imputed, with the exception of the following parameters: fidaxomicin start date, assumed to be the fidaxomicin prescription date; fidaxomicin stop date, set as the start date plus 10 days; incomplete CDI dates with only month and year present, in which case the start date was set to the first day of the month and the stop date to the last day of the month; start date of prior/concomitant or post-fidaxomicin medication, which was set to the first day of the month and/or January if either day or month was missing, or assumed to have started prior to first fidaxomicin dose if completely missing; stop date of prior/concomitant or post-fidaxomicin medication, which was set to the last day of the month and/or December if either day or month was missing; ECG or laboratory assessments at baseline, in which case data at admission were used; ECG or laboratory assessments at the end of the observation period, in which case data at time of last fidaxomicin dose were used.

#### Prior and concomitant antibacterial use

Coding of medical terms and medications was performed using the Medical Dictionary for Regulatory Activities (MedDRA), version 18.0, and the World Health Organization Drug Dictionary (WHO-DD), March 2015, respectively. Prior antibacterial use was defined as antibacterials taken within the 30 days prior to or on the day of start of fidaxomicin treatment. Antibacterials stopping on the first date of fidaxomicin treatment were defined as previous medications. Concomitant medication was defined as medication taken any time from on or after the first dose date to the last dose date of fidaxomicin.

#### Laboratory parameters

Systematic errors in laboratory parameter units were identified following database lock. These values were corrected using the rules presented in Supplementary Table 2 and the corrected values used for analysis. In addition, extremely high and low values outside of biologically plausible ranges (Supplementary Table 3) were excluded from analyses.

#### Quality control

To enhance data quality, the electronic case report forms included programmable edits to obtain immediate feedback if data were missing, out of range, illogical or potentially erroneous. Concurrent manual data review was also performed and any queries generated within the electronic data capture system followed up for resolution. The data were reviewed on a regular basis by the Contract Research Organisation data manager and the study sponsor medical reviewer.

##### Data availability

Access to anonymized individual patient-level data will not be provided for this trial as it meets one or more of the exceptions described under the Sponsor Specific Information for Astellas on www.clinicalstudydatarequest.com.

## Results

### Patient characteristics

Of 582 patients enrolled at 22 sites, six patients were excluded from the analysis due to being outside the country’s eligibility period (four patients), initial misunderstanding at the investigational site (one patient) and complete lack of follow-up data (one patient). Data from 576 patients were therefore analysed, corresponding to 590 treatment episodes. The majority of patients (564/576, 97.9%) had one treatment episode, and for almost all treatment episodes (569/590, 96.4%), there was only one fidaxomicin prescription (Supplementary Table 4). CDI was confirmed in 97.2% (519/534) of treatment episodes for which data were available (Table 2). In the 3 months preceding the most recent treatment episode, 23.9% (141/590) patients had experienced a previous CDI occurrence (Supplementary Table 5).

**Table 2:** CDI confirmation for the most recent treatment episode

	IBD (N = 29)	Fulminant CDI-PI (N = 88)	Fulminant CDI-SS (N = 119)	Moderate-to-severe hepatic impairment (N = 51)	Severe renal impairment (N = 109)	No MCSI (N = 319)	Total (N = 590)
CDI duration (days)							
n	16	64	89	41	57	205	385
Median (min, max)	11.5 (3, 60)	14.0 (4, 180)	13.0 (2, 180)	12.0 (4, 55)	11.0 (2, 85)	10.0 (2, 126)	12.0 (2, 180)
CDI was objectively confirmed, n (%)							
n	27	82	111	46	96	289	534
Yes	26 (96.3)	77 (93.9)	107 (96.4)	44 (95.7)	95 (99.0)	282 (97.6)	519 (97.2)
If yes, CDI confirmation method^a^, n (%)							
n	26	77	107	44	95	282	519
PCR	14 (53.8)	36 (46.8)	37 (34.6)	17 (38.6)	29 (30.5)	84 (29.8)	171 (32.9)
Toxin detection	18 (69.2)	48 (62.3)	75 (70.1)	32 (72.7)	77 (81.1)	223 (79.1)	402 (77.5)
Culture	12 (46.2)	31 (40.3)	29 (27.1)	14 (31.8)	28 (29.5)	77 (27.3)	151 (29.1)
Other	3 (11.5)	3 (3.9)	13 (12.1)	8 (18.2)	12 (12.6)	40 (14.2)	68 (13.1)

As some patients presented with > 1 MCSI, the sum of the number of patients with each MCSI is greater than the total number of patients. In the event of a patient having more than one treatment episode with fidaxomicin, treatment episodes are considered distinct if separated by more than 30 days from last dose of the earlier treatment episode to the first dose of the subsequent treatment episode. Statistics and percentages are based on the total number of treatment episodes with known data (excluding missing and unknown data)

*CDI Clostridium difficile* infection, *IBD* inflammatory bowel disease, *PCR* polymerase chain reaction, *MCSI* medical condition of specific interest, *N* number of treatment episodes, *n* number of observations with known data, *PI* principal investigator, *SS* scoring system

^a^Multiple diagnostic methods were often used simultaneously

### Proportion of patients with an MCSI

Patients with at least one MCSI represented 45.3% (261/576) of the study population and had 45.9% (271/590) of treatment episodes (Supplementary Table 4). Patient characteristics by MCSI are shown in Supplementary Table 4. No pregnancy was reported.

### Deaths

**Fig. 2:**
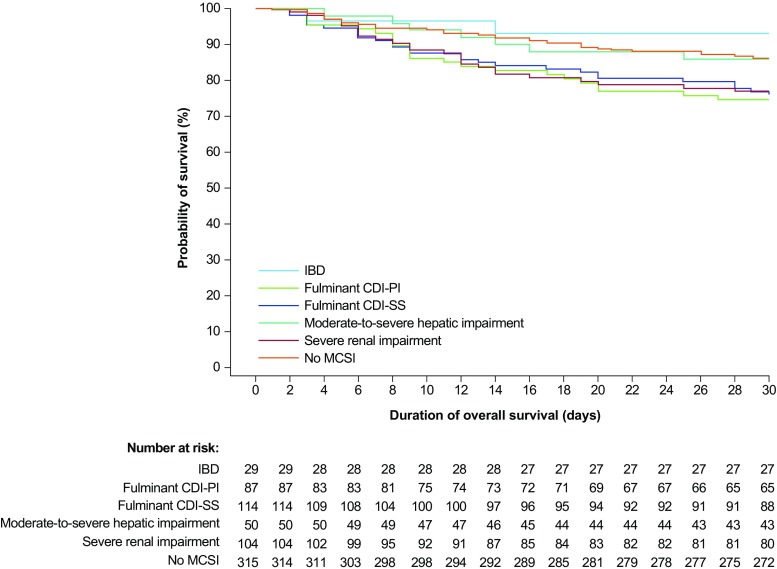
Kaplan-Meier plot of overall 30-day survival by MCSI. The 30-day period starts on day 1 of fidaxomicin treatment. CDI *Clostridium difficile* infection, IBD inflammatory bowel disease, MCSI medical condition of specific interest, PI principal investigator, SS scoring system

Overall, 17.0% (98/576) of patients died within 30 days of the first fidaxomicin dose of the most recent treatment episode (30-day mortality) (Table 3). Of those with information about the cause of death, 29.6% (24/81) of deaths were attributed to CDI (Table 3). In the total study population, CDI-related mortality was 5.0% across all subgroups and lowest (1.9%) in patients without MCSIs (Table 3; percentages adjusted for missing values). There was a trend for patients with fulminant CDI-PI, fulminant CDI-SS or severe renal impairment to have the poorest 30-day survival rates (Fig. 2).

**Table 3:** Deaths of patients with CDI within 30 days of first dose of fidaxomicin (first fidaxomicin dose of the most recent treatment period) by MCSI

	IBD (N = 29)	Fulminant CDI-PI (N = 87)	Fulminant CDI-SS (N = 114)	Moderate-to-severe hepatic impairment (N = 50)	Severe renal impairment (N = 104)	No MCSI (N = 315)	Total (N = 576)
Deaths, n (%)	2 (6.9)	24 (27.6)	28 (24.6)	7 (14.0)	27 (26.0)	44 (14.0)	98 (17.0)
Cause of death related to CDI, n (%)							
n	2	20	23	6	23	37	81
Yes	2 (100.0)	11 (55.0)	11 (47.8)	2 (33.3)	9 (39.1)	5 (13.5)	24 (29.6)
Estimate of all patients in whom cause of death is CDI-related (%)^a^							
CDI-related death in population (adjusted for unknown)	6.9%	15.2%	11.7%	4.7%	10.2%	1.9%	5.0%

As some patients presented with > 1 MCSI, the sum of the number of patients with each MCSI is greater than the total number of patients. Statistics and percentages are based on the total number of treatment episodes with known data (excluding missing and unknown data)

*CDI Clostridium difficile* infection, *IBD* inflammatory bowel disease, *MCSI* medical condition of specific interest, *N* number of patients, *n* number of patients with known data, *PI* principal investigator, *SS* scoring system

^a^The percentage of all patients where the cause of death is related to CDI, adjusted for missing causality assessment, is calculated by multiplying the overall percentage of deaths by the percentage of deaths related to CDI

### Changes in laboratory parameters

In the overall patient population, median haemoglobin values were below the normal range of 140–175 g/L throughout the study (Table 4), with no apparent changes attributable to fidaxomicin. Median leucocyte counts for the overall population were within the normal range of 4.5–11 × 10^9^/L throughout the study; a decrease in leucocyte counts was observed in the majority of MCSI subgroups following fidaxomicin treatment. Liver function parameters, which were in line with patients’ underlying medical conditions, and renal function parameters underwent no apparent changes attributable to fidaxomicin at the study population level. Of note, the availability of laboratory test results decreased substantially over the observational period across all subgroups, most probably as the result of a reduced medical need for collecting such data; the numbers of patients with both baseline and post-treatment data were therefore low.

**Table 4:** Laboratory test results by MCSI

Parameter, median (min, max)	IBD (N = 29)	Fulminant CDI-PI (N = 87)	Fulminant CDI-SS (N = 114)	Moderate-to-severe hepatic impairment (N = 50)	Severe renal impairment (N = 104)	No MCSI (N = 315)	Total (N = 576)
Serum albumin (g/L)							
Baseline	28.0 (18, 43) n = 15	25.5 (2, 45) n = 30	26.0 (2, 25) n = 53	27.0 (18, 38) n = 34	26.0 (13, 43) n = 50	28.9 (14, 58) n = 136	27.0 (2, 58) n = 267
End of treatment	32.0 (18, 40) n = 11	28.0 (14, 44) n = 18	27.0 (13, 66) n = 44	29.5 (6, 41) n = 22	25.0 (6, 42) n = 33	28.0 (16, 43) n = 78	29.0 (6, 66) n = 165
End of observation period	30.0 (17, 49) n = 7	34.0 (17, 40) n = 12	31.0 (17, 40) n = 22	34.0 (15, 48) n = 14	33.0 (20, 51) n = 17	33.0 (15, 46) n = 49	33.0 (15, 51) n = 101
Alanine aminotransferase (μkat/L)							
Baseline	0.259 (0.07, 1.20) n = 12	0.317 (0.05, 1.65) n = 52	0.409 (0.05, 3.66) n = 64	0.434 (0.12, 2.52) n = 31	0.184 (0.00, 3.66) n = 46	0.267 (0.05, 4.94) n = 117	0.284 (0.00, 4.94) n = 248
End of treatment	0.292 (0.10, 1.40) n = 8	0.408 (0.12, 2.44) n = 27	0.434 (0.12, 5.98) n = 37	0.610 (0.12, 4.88) n = 16	0.334 (0.10, 5.98) n = 25	0.267 (0.08, 1.14) n = 63	0.334 (0.08, 5.98) n = 139
End of observation period	0.484 (0.15, 2.36) n = 5	0.276 (0.10, 1.00) n = 16	0.242 (0.10, 1.05) n = 22	0.351 (0.10, 2.72) n = 11	0.204 (0.05, 0.62) n = 15	0.401 (0.13, 2.57) n = 29	0.334 (0.05, 2.72) n = 73
Aspartate aminotransferase (μkat/L)							
Baseline	0.251 (0.13, 1.40) n = 13	0.384 (0.13, 1.42) n = 47	0.459 (0.13, 1.70) n = 54	0.718 (0.17, 2.61) n = 35	0.418 (0.15, 1.35) n = 33	0.301 (0.12, 12.03) n = 95	0.367 (0.12, 12.03) n = 208
End of treatment	0.334 (0.07, 1.52) n = 9	0.359 (0.10, 2.45) n = 26	0.518 (0.10, 2.45) n = 39	0.726 (0.10, 12.24) n = 22	0.551 (0.13, 1.29) n = 23	0.392 (0.15, 33.17) n = 54	0.443 (0.07, 33.17) n = 128
End of observation period	0.234 (0.15, 0.43) n = 6	0.251 (0.17, 1.02) n = 15	0.251 (0.17, 2.35) n = 21	0.401 (0.17, 2.29) n = 15	0.251 (0.17, 0.65) n = 13	0.434 (0.18, 46.60) n = 39	0.376 (0.15, 46.60) n = 86
Serum creatinine (μmol/L)							
Baseline	69.0 (18, 309) n = 19	84.0 (18, 556) n = 73	108.0 (18, 964) n = 103	82.2 (18, 898) n = 44	237.6 (54, 964) n = 88	64.8 (5, 376) n = 228	76.9 (5, 964) n = 446
End of treatment	56.0 (18, 336) n = 14	73.0 (18, 460) n = 47	80.0 (18, 751) n = 83	96.2 (18, 996) n = 26	183.4 (27, 782) n = 62	62.4 (6, 493) n = 118	76.0 (6, 996) n = 267
End of observation period	49.0 (18, 168) n = 9	61.9 (18, 281) n = 21	67.2 (18, 469) n = 37	71.0 (18, 401) n = 17	157.5 (38, 646) n = 26	65.0 (1, 178) n = 63	76.0 (1, 646) n = 139
Total bilirubin (μmol/L)							
Baseline	8.5 (3, 38) n = 16	10.2 (3, 270) n = 52	9.5 (2, 185) n = 74	20.3 (6, 277) n = 40	6.7 (2, 51) n = 54	7.0 (2, 487) n = 147	8.0 (2, 487) n = 301
End of treatment	6.0 (2, 214) n = 13	7.0 (2, 407) n = 33	6.8 (2, 407) n = 54	17.0 (4, 445) n = 25	6.0 (2, 214) n = 37	6.0 (1, 82) n = 85	6.8 (1, 445) n = 190
End of observation period	6.0 (3, 17) n = 8	6.8 (3, 15) n = 18	6.8 (3, 159) n = 28	14.9 (4, 36) n = 15	6.0 (2, 36) n = 19	6.0 (2, 181) n = 53	6.8 (2, 181) n = 112
Serum lactate (mmol/L)							
Baseline	0.52 (0.5, 0.5) n = 1	1.12 (0.5, 4.2) n = 14	1.12 (0.5, 4.2) n = 22	1.65 (0.6, 3.3) n = 10	1.04 (0.5, 4.2) n = 6	1.10 (0.2, 3.3) n = 13	1.30 (0.2, 4.2) n = 41
End of treatment	0.45 (0.5, 0.5) n = 1	0.61 (0.5, 5.4) n = 7	0.61 (0.5, 5.4) n = 9	0.92 (0.5, 5.4) n = 4	0.53 (0.5, 0.6) n = 2	0.80 (0.8, 0.8) n = 1	0.71 (0.5, 5.4) n = 10
End of observation period	0.72 (0.7, 0.7) n = 1	0.69 (0.6, 0.8) n = 4	0.69 (0.6, 0.8) n = 4	0.82 (0.8, 0.8) n = 1	0.72 (0.7, 0.7) n = 1	0.62 (0.6, 0.6) n = 1	0.65 (0.6, 0.8) n = 5
Haemoglobin (g/L)							
Baseline	103.0 (74, 152) n = 21	95.0 (41.0, 183) n = 73	95.5 (41, 181) n = 102	96.0 (70, 140) n = 42	95.0 (41, 183) n = 83	99.0 (64, 151) n = 223	98.0 (41, 183) n = 436
End of treatment	90.0 (73, 150) n = 14	96.0 (73, 133) n = 49	97.0 (68, 133) n = 82	91.5 (68, 117) n = 26	97.0 (53, 146) n = 60	98.5 (42, 159) n = 120	97.0 (42, 159) n = 268
End of observation period	94.0 (86, 155) n = 9	93.0 (23, 810) n = 21	92.0 (68, 810) n = 37	92.5 (61, 121) n = 18	97.0 (81, 150) n = 27	99.5 (68, 162) n = 60	98.0 (23, 810) n = 137
Leucocytes (× 10^9^/L)							
Baseline	7.30 (1.5, 36.3) n = 21	9.20 (0.1, 49.1) n = 73	14.25 (0.0, 603.0) n = 103	7.08 (1.1, 42.3) n = 42	10.11 (2.2, 39.4) n = 83	8.40 (0.0, 90.1) n = 223	8.76 (0.0, 603.0) n = 437
End of treatment	9.05 (0.9, 24.4) n = 14	8.30 (0.1, 30.7) n = 49	9.79 (0.6, 48.2) n = 82	6.20 (0.9, 25.1) n = 26	9.23 (0.6, 33.6) n = 60	8.35 (0.0, 24.4) n = 118	8.41 (0.0, 48.2) n = 266
End of observation period	7.25 (2.2, 18.7) n = 9	7.30 (2.3, 17.3) n = 21	7.90 (0.2, 17.3) n = 37	5.10 (2.0, 14.0) n = 18	6.80 (3.9, 14.1) n = 27	7.75 (0.0, 36.8) n = 60	7.30 (0.0, 36.8) n = 137

Laboratory correction rules were applied to some laboratory parameters to correct systematic errors in unit and the exclusion of values outside of biologically feasible ranges; parameters affected are creatinine, haemoglobin, haematocrit, serum albumin, serum lactate and total bilirubin. As some patients presented with > 1 MCSI, the sum of the number of patients with each MCSI is greater than the total number of patients. In the event of a patient having more than one treatment episode with fidaxomicin, treatment episodes are considered distinct if separated by more than 30 days from last dose of the earlier treatment episode to the first dose of the subsequent treatment episode. Statistics are based on the total number of treatment episodes with known data (excluding missing and unknown data)

*CDI Clostridium difficile* infection, *IBD* inflammatory bowel disease, *MCSI* medical condition of specific interest, *N* number of patients, *n* number of patients with known data, *PI* principal investigator, *SS* scoring system

### Changes in ECG findings

In the total population, ECG results were available for 13.0% (75/576) of patients at admission, decreasing to 4.0% (23/576) of patients at the end of the observational period (Supplementary Table 6), possibly as they were performed only on the basis of medical need. Across all subgroups, clinically significant abnormal ECGs represented 22.7% (17/75) of assessments at baseline, reducing to 4.3% (1/23) of assessments at the end of the observational period. However, numbers of patients with both baseline and post-treatment data were low.

### Fidaxomicin exposure

Overall, 611 prescriptions were issued in 590 fidaxomicin treatment episodes. Adherence to the fidaxomicin dosing schedule was as recommended (200 mg twice daily for 10 days) in 73.1% (431/590) of treatment episodes (Supplementary Table 7). The regimen was completed by most patients (77.9%, 457/587); reasons for discontinuation included death (4.4%, 26/590 treatment episodes), followed by lack of efficacy (1.2%, 7/587) and adverse event (0.7%, 4/587). No particular reasons for discontinuation were associated with any MCSI.

### Fidaxomicin response

In the overall population, diarrhoea resolved in 78.0% (404/518) of fidaxomicin treatment episodes, with a median time to resolution of 6.0 days (Table 5). Resolution of diarrhoea was lowest in patients with fulminant CDI-PI (67.5%, 56/83), fulminant CDI-SS (68.9%, 73/106) and severe renal impairment (68.0%, 68/100).

**Table 5:** Fidaxomicin response by treatment episode and MCSI

	IBD (N = 29)	Fulminant CDI-PI (N = 88)	Fulminant CDI-SS (N = 119)	Moderate-to-severe hepatic impairment (N = 51)	Severe renal impairment (N = 109)	No MCSI (N = 319)	Total (N = 590)
Resolution of diarrhoea, n (%)							
n	22	83	106	47	100	275	518
Yes	18 (81.8)	56 (67.5)	73 (68.9)	37 (78.7)	68 (68.0)	229 (83.3)	404 (78.0)
Time to resolution^a^, days							
n	16	52	69	36	51	193	347
Median (min, max)	6.5 (2, 32)	5.0 (2, 32)	7.0 (1, 38)	5.0 (2, 13)	8.0 (1, 43)	5.0 (1, 367^c^)	6.0 (1, 367^c^)
30-day recurrence, n (%)							
n	21	72	86	38	78	221	420
Yes	4 (19.0)	10 (13.9)	15 (17.4)	7 (18.4)	14 (17.9)	41 (18.6)	79 (18.8)
Time to recurrence^b^, days							
n	2	9	13	7	11	37	68
Median (min, max)	11.5 (10, 13)	19.0 (15, 47^c^)	19.0 (6, 44^c^)	24.0 (6, 28)	19.0 (3, 31^c^)	17.0 (6, 39^c^)	18.5 (3, 47^c^)

As some patients presented with > 1 MCSI, the sum of the number of patients with each MCSI is greater than the total number of patients. Statistics and percentages are based on the total number of treatment episodes with known data (excluding missing and unknown data)

*CDI Clostridium difficile* infection, *IBD* inflammatory bowel disease, *MCSI* medical condition of specific interest, *N* number of treatment episodes, *n* number of observations with known data, *PI* principal investigator, *SS* scoring system

^a^From first dose of fidaxomicin, for patients who experienced resolution of diarrhoea

^b^From last dose of fidaxomicin

^c^As reported; likely an outlier

Recurrence of diarrhoea within 30 days after end of treatment occurred following 18.8% (79/420) of treatment episodes in the study population and was similar for most MCSI subgroups (Table 5). Median time to recurrence in the total population was 18.5 days (range 3–47 days); this was similar across subgroups with the exception of patients with IBD for whom the median time to recurrence was 11.5 days (two observations).

### Prior and concomitant antibacterial use

The majority of patients (87.3%, 503/576) in the study population received antibacterials within 30 days preceding fidaxomicin treatment, the most frequent being metronidazole (43.1%, 248/576) and vancomycin (41.7%, 240/576) (Table 6). Concomitantly with fidaxomicin treatment, 58.2% (335/576) of patients received other antibacterials; patients with moderate-to-severe hepatic impairment had the highest level of concomitant antibacterial use (72.0%, 36/50) (Table 6).

**Table 6:** Prior and concomitant use of antibacterials by MCSI

	IBD (N = 29)	Fulminant CDI-PI (N = 87)	Fulminant CDI-SS (N = 114)	Moderate-to-severe hepatic impairment (N = 50)	Severe renal impairment (N = 104)	No MCSI (N = 315)	Total (N = 576)
Prior antibacterial use^a^, n (%)							
Any antibacterial	22 (75.9)	79 (90.8)	107 (93.9)	44 (88.0)	92 (88.5)	271 (86.0)	503 (87.3)
Metronidazole	13 (44.8)	53 (60.9)	54 (47.4)	25 (50.0)	42 (40.4)	130 (41.3)	248 (43.1)
Vancomycin	10 (34.5)	48 (55.2)	54 (47.4)	25 (50.0)	34 (32.7)	130 (41.3)	240 (41.7)
Piperacillin/enzyme inhibitor	4 (13.8)	22 (25.3)	44 (38.6)	10 (20.0)	31 (29.8)	88 (27.9)	161 (28.0)
Meropenem	2 (6.9)	33 (37.9)	37 (32.5)	20 (40.0)	19 (18.3)	52 (16.5)	119 (20.7)
Amoxicillin/ enzyme inhibitor	1 (3.4)	11 (12.6)	28 (24.6)	6 (12.0)	19 (18.3)	50 (15.9)	97 (16.8)
Ciprofloxacin	4 (13.8)	12 (13.8)	12 (10.5)	9 (18.0)	12 (11.5)	37 (11.7)	69 (12.0)
Gentamicin	1 (3.4)	6 (6.9)	23 (20.2)	3 (6.0)	13 (12.5)	32 (10.2)	64 (11.1)
Levofloxacin	2 (6.9)	10 (11.5)	11 (9.6)	4 (8.0)	8 (7.7)	19 (6.0)	41 (7.1)
Teicoplanin	1 (3.4)	7 (8.0)	9 (7.9)	7 (14.0)	5 (4.8)	14 (4.4)	32 (5.6)
Trimethoprim/sulfamethoxazole	1 (3.4)	2 (2.3)	5 (4.4)	9 (18.0)	5 (4.8)	12 (3.8)	28 (4.9)
Linezolid	0	3 (3.4)	4 (3.5)	6 (12.0)	1 (1.0)	10 (3.2)	21 (3.6)
Concomitant antibacterial use^b^, n (%)							
Any antibacterial	14 (48.3)	56 (64.4)	73 (64.0)	36 (72.0)	66 (63.5)	172 (54.6)	335 (58.2)
Piperacillin/ enzyme inhibitor	2 (6.9)	9 (10.3)	20 (17.5)	6 (12.0)	20 (19.2)	47 (14.9)	85 (14.8)
Metronidazole	6 (20.7)	17 (19.5)	20 (17.5)	12 (24.0)	14 (13.5)	33 (10.5)	75 (13.0)
Meropenem	2 (6.9)	14 (16.1)	21 (18.4)	11 (22.0)	16 (15.4)	40 (12.7)	80 (13.9)
Vancomycin	1 (3.4)	17 (19.5)	19 (16.7)	8 (16.0)	11 (10.6)	30 (9.5)	64 (11.1)
Ciprofloxacin	0	5 (5.7)	5 (4.4)	5 (10.0)	2 (1.9)	15 (4.8)	26 (4.5)
Linezolid	0	2 (2.3)	5 (4.4)	6 (12.0)	3 (2.9)	12 (3.8)	24 (4.2)
Trimethoprim/sulfamethoxazole	1 (3.4)	1 (1.1)	3 (2.6)	7 (14.0)	4 (3.8)	9 (2.9)	21 (3.6)

As some patients presented with > 1 MCSI, the sum of the number of patients with each MCSI is greater than the total number of patients. Statistics and percentages are based on the total number of treatment episodes with known data (excluding missing and unknown data). Antibacterials included are those used by ≥ 10% patients within any MCSI subgroup

*CDI Clostridium difficile* infection, *IBD* inflammatory bowel disease, *MCSI* medical condition of specific interest, *N* number of patients, *n* number of patients with known data, *PI* principal investigator, *SS* scoring system

^a^Systemic antibacterials taken ≤ 30 days before the date of first fidaxomicin dosing (inclusive) of the first treatment episode

^b^Systemic antibacterials taken any time from the first fidaxomicin dosing date to the last dose of fidaxomicin, of the first treatment episode

## Discussion

The ANEMONE study, a post-approval fidaxomicin utilisation study in a routine clinical setting, presented a substantial proportion (45.3%) of patients with MCSIs, such as fulminant or life-threatening CDI, severe renal impairment, moderate-to-severe hepatic impairment and IBD. Limited data on fidaxomicin use are available for these patient groups, particularly for patients with fulminant or life-threatening CDI and/or IBD who were excluded from fidaxomicin phase III trials. Consequently, caution is currently advised when treating these patients with fidaxomicin. This study provides important data regarding safety and effectiveness of fidaxomicin in these at-risk patient populations.

The 30-day mortality rate of 17.0% in this study population was within the range of rates observed in other in-hospital studies of CDI (9–38%) [17]. The CDI attributable death rate in the whole study population (5.0%) was also similar to the 6.0% CDI attributable mortality rate in a pooled analysis of 10,975 patients from 27 studies [18]. However, caution should be used when comparing these data due to the different populations and analysis methods used in the studies. Additionally, there were no changes in ECG or laboratory parameters that could be ascribed to fidaxomicin, with the exception of a decrease in leucocyte counts in the majority of patients with MCSIs and also in the overall population, consistent with a positive response to treatment.

In our study, resolution of diarrhoea occurred for a slightly lower proportion (78.0%) of treatment episodes than in two previous phase III trials (87.7 and 88.2%) [11, 12] and a retrospective Spanish study (90.3%) [19], but was similar to that in a previous US study (77.4%) [20]. For patients with IBD, a known risk factor for CDI [21], our study found that resolution of diarrhoea occurred in a similar proportion of treatment episodes (81.8%) to that for patients without any MCSI (83.3%). In comparison, a previous open-label study of fidaxomicin reported that resolution of diarrhoea occurred in 90% of the overall patient population but 100% of patients with IBD [22].

The incidence of 30-day recurrence in our study (18.8%) was slightly higher than that observed during a 28-day follow-up period in the phase III trials (12.7 and 15.4%) [11, 12] and similar to that in a Spanish study (16.7%), which included a large proportion of patients with comorbidities and/or severe CDI [19]. A retrospective study of hospitalised patients in the USA reported a 90-day recurrence rate of 21% and included patients with renal disease (29%), diabetes (28%), gastrointestinal disorders (25%) and cancers (26%), all of whom had prior CDI episodes treated with metronidazole and/or vancomycin [20]. In a study of patients with chronic kidney disease (CKD), recurrence was 16% for those with no CKD, increasing to 24% for those with stage 4 CKD [23]. By contrast, incidence of recurrence did not increase with the presence of an MCSI in this study.

The majority of patients in our study received other antibacterials either sequentially (≤ 30 days prior) or concomitantly with fidaxomicin, which is similar to other studies of fidaxomicin routine clinical use [20, 24]. The high number of patients who had received prior metronidazole (43.1%) or vancomycin (41.7%) may also be reflective of the use of fidaxomicin predominantly in patient populations in whom previous lines of therapy have failed. In light of this, the observed rate of diarrhoea resolution (78.0%) therefore appears particularly positive. However, the use of fidaxomicin only as a second-line or concomitant medication may reduce its comparative benefits: a previous study of UK hospitals found that the reduction in recurrence was greater when fidaxomicin was used as a first-line therapy in all CDI episodes than when fidaxomicin was used in only selected cases, such as in patients with recurrence or at risk of recurrence [25].

The retrospective design enabled the study of routine clinical practice by including centres with previously available fidaxomicin prescription data. Selection bias was reduced by exclusively enrolling patients who had been treated prior to the investigator’s decision to participate and limiting recruitment numbers to a random 50 patients per site. However, meaningful comparisons among the individual patient groups were problematic as a number of patients had more than one MCSI. Moreover, the fulminant CDI-SS subgroup incorporated a greater number of patients than fulminant CDI-PI, and some minor differences in outcomes were observed between these patient groups. Finally, any causal assessment of safety or efficacy outcomes in this study was limited by the non-interventional study design. Results for ECG and laboratory parameters should be interpreted with caution due to a paucity of follow-up data, reflecting this real-world setting where follow-up measurements are often considered redundant after a patient’s recovery.

In conclusion, this study found that, in routine clinical practice, almost half of patients receiving fidaxomicin had MCSIs. This finding highlights the need for further prospective studies in these patient populations, who are often excluded from clinical trials yet are highly represented within the fidaxomicin-treated CDI population. Irrespective of the presence of MCSIs, the majority of patients had a positive response to treatment as measured by resolution of diarrhoea. No particular safety signals were observed in these patient groups.

## Electronic supplementary material

ESM 1 (DOCX 56 kb)
