# Correction to: Fidaxomicin for the treatment of *Clostridium difficile* infection (CDI) in at-risk patients with inflammatory bowel disease, fulminant CDI, renal impairment or hepatic impairment: a retrospective study of routine clinical use (ANEMONE)

**DOI:** 10.1007/s10096-018-3410-8

**Published:** 2018-11-08

**Authors:** Maria J. G. T. Vehreschild, Surabhi Taori, Simon D. Goldenberg, Florian Thalhammer, Emilio Bouza, Joop van Oene, Graham Wetherill, Areti Georgopali

**Affiliations:** 1 Department I of Internal Medicine, University Hospital of Cologne and German Centre for Infection Research, Partner Site Bonn-Cologne, Cologne, Germany; 2 King’s College Hospital NHS Foundation Trust, London, UK; 3 King’s College London & Guy’s and St Thomas’ NHS Foundation Trust, London, UK; 4 Department of Infectious Diseases and Tropical Medicine, Division of Internal Medicine I, Medical University of Vienna, Vienna, Austria; 5 Clinical Microbiology and Infectious Diseases, Hospital Gregorio Marañón, Madrid, Spain; 6 Department of Medicine, Ciber de Enfermedades Respiratorias (CIBERES), Complutense University, Madrid, Spain; 7 Astellas Pharma Europe B.V, Leiden, The Netherlands; 8 Astellas Pharma, Inc., Chertsey, UK

**Correction to: European Journal of Clinical Microbiology & Infectious Diseases (2018) 37:2097–2106**

10.1007/s10096-018-3344-1

The article “Fidaxomicin for the treatment of Clostridium difficile infection (CDI) in at-risk patients with inflammatory bowel disease, fulminant CDI, renal impairment or hepatic impairment: a retrospective study of routine clinical use (ANEMONE)”, written by Maria J. G. T. Vehreschild, Surabhi Taori, Simon D. Goldenberg, Florian Thalhammer, Emilio Bouza, Joop van Oene, Graham Wetherill, and Areti Georgopali, was originally published electronically on the publisher’s internet portal SpringerLink on 11 August 2018 without open access.

With the authors’ decision to opt for Open Choice the copyright of the article changed on October 2018 to © The Author(s) 2018 and the article is forthwith distributed under the terms of the Creative Commons Attribution 4.0 International License (http://creativecommons.org/licenses/by/4.0/), which permits use, duplication, adaptation, distribution and reproduction in any medium or format, as long as you give appropriate credit to the original author(s) and the source, provide a link to the Creative Commons license, and indicate if changes were made.

The original article has been corrected.

