# Supplementary material for: Fidaxomicin for the treatment of Clostridium difficile infection (CDI) in at-risk patients with inflammatory bowel disease, fulminant CDI, renal impairment or hepatic impairment: a retrospective study of routine clinical use (ANEMONE)
Source: Eur J Clin Microbiol Infect Dis. 2018 Aug 11;37(11):2097–106. doi: 10.1007/s10096-018-3344-1 (PMC6315004; doi:10.1007/s10096-018-3344-1)
Supplement: Supplementary file 1 — (DOCX 56 kb) [file 10096_2018_3344_MOESM1_ESM.docx]

Supplementary information for the manuscript entitled ‘Fidaxomicin for the treatment of *Clostridium difficile* infection (CDI) in at-risk patients with inflammatory bowel disease, fulminant CDI, renal impairment or hepatic impairment: a retrospective study of routine clinical use (ANEMONE)’

**Authors:** Maria JGT Vehreschild, Surabhi Taori, Simon D Goldenberg, Florian Thalhammer, Emilio Bouza, Joop van Oene, Graham Wetherill, Areti Georgopali

# Supplementary Tables

# Supplementary Table 1 Scoring system used to define fulminant CDI

| **Parameter evaluation** | **Points** | **If patient shows ≥1 of the following conditions** |
| --- | --- | --- |
| Abdominal pain and/or distention | 1 | - Abdominal cramping/pain in current CDI occurrence |
| Hypoalbuminemia (<3 g/dL) | 1 | - Serum albumin <3 g/dL in ‘laboratory findings’ - Serum albumin in ‘laboratory assessments’ <3 g/dL at admission, last assessment before first fidaxomicin dose, or time of last fidaxomicin dose |
| Fever | 1 | - Fever in ‘physical exam’ - Fever in ‘current CDI episode’, as determined by physical examination |
| ICU at admission | 1 | - Patient admitted to ICU in ‘admission and infection history’ before or at the time of fidaxomicin treatment |
| CT scan with non-specific findings of pancolitis, ascites and/or bowel wall thickening | 2 | - Patient with distention, colonic wall, pericolonic, and/or ascites in ‘imaging results’ |
| WBC count >15,000/μL, or <1,500/μL and/or band count >10% | 2 | - Increased WBC count in current CDI occurrence - Leukocytes >15×10^9^/L in ‘laboratory findings’ - WBC in ‘laboratory assessments’ >1500/μL or <1500/μL at admission, last assessment before first fidaxomicin dose, or time of last fidaxomicin dose |
| Creatinine 1.5 fold >baseline | 2 | - Serum creatinine >50% above baseline in ‘laboratory findings’ - Serum creatinine ≥133 μmol/L in ‘laboratory findings’ |
| Abdominal peritoneal signs | 3 | - Signs and symptoms of peritonitis in ‘physical exam’ |
| Vasopressors required | 5 | - Haemodynamic instability in current CDI occurrence - Haemodynamic instability, including signs of distributive shock, in ‘physical exam’ |
| Mechanical ventilation required  – attributed to CDI | 5 | - Respiratory failure requiring mechanical ventilation in ‘physical exam’ |

CT, computerised tomography; CDI, *Clostridium difficile* infection; ICU, intensive care unit; WBC, white blood cell. The following parameters do not take part of the final score: immunosuppression and/or chronic medical condition and disorientation, confusion or decreased consciousness. Patients with a final score ≥7 points were considered to have fulminant CDI. Adapted from Van der Wilden et al. J Trauma Acute Care Surg. 2013;76(2):424-430

# **Supplementary Table 2** Rules for correction of unit error in laboratory values

| **Laboratory parameter** | **Rule** |
| --- | --- |
| Haemoglobin | If haemoglobin >99 g/dL, then change unit into g/L  If haemoglobin <20 g/L then change unit into g/dL |
| Haematocrit | If haematocrit <1% then change unit into L/L |
| Serum albumin | If albumin ≥10 g/dL then divide by a factor 10 and keep the unit as g/dL |
| Creatinine | If unit of creatinine is mmol/L then change to μmol/L |
| Total bilirubin | If unit of total bilirubin is mmol/L then change to μmol/L |

**Supplementary Table 3** Biologically feasible ranges by laboratory parameter

| **Laboratory**  **parameter** | **SI unit** [26–30] | **Observed**  **range (SI unit)** | **Conventional**  **unit** | **Biologically feasible**  **ranges** [31] |
| --- | --- | --- | --- | --- |
| Alanine aminotransferase | 0.17–0.68 µkat/L | 0–7.3647 | 10–40 U/L | N/A |
| Aspartate aminotransferase | 0.17–0.51 µkat/L | 0.0668–12.2411 | 10–30 U/L | N/A |
| Urea | 2.9–8.2 mmol/L | 0.6–99 | 8–23 mg/dL | N/A |
| Haematocrit | 0.41–0.50 L/L | 0.0021–0.53 | 41–50% | 0.10–0.90 L/L |
| Haemoglobin | 140–175 g/L | 7.3–1330 | 14.0–17.5 g/dL | 20–250 g/L |
| Neutrophils | 0–0.7 x 10^9^/L | 0–85.5 | 1800–8500/µL | N/A |
| Serum albumin | 32–56 g/L | 2–520 | 3.5–5.0 g/dL | <60 g/L |
| Serum creatinine | 53–106 µmol/L | 1.16–1401.14 | 0.6–1.2 mg/dL | N/A |
| Serum lactate | 0.6–1.7 mmol/L | 0.1221–7.5 | 5–15 mg/dL | <47.7 mmol/L [32] |
| Total bilirubin | 5–21 µmol/L | 1–486.95088 | 0.3–1.2 mg/dL | N/A |
| Leucocyte  count | 4.5–11 x 10^9^/L | 0–603 | 4500–11000/µL | N/A |

N/A, no biologically plausible range exists for the laboratory parameter; SI, system international

**Supplementary Table 4** Patient demographic and clinical characteristics by MCSI

|  | **IBD** | **Fulminant CDI-PI** | **Fulminant CDI-SS** | **Moderate-to-severe hepatic impairment** | **Severe renal impairment** | **No MCSI** | **Total** |
| --- | --- | --- | --- | --- | --- | --- | --- |
| **Patients, n (%), 95% CI** | 29 (5.0)  3.4–7.2 | 87 (15.1)  12.3–18.3 | 114 (19.8)  16.6–23.3 | 50 (8.7)  6.5–11.3 | 104 (18.1)  15.0–21.4 | 315 (54.7)  50.5–58.8 | 576 (100.0) |
| **Age (years), median (min, max)** | 64.0 (20, 97) | 71.0 (23, 90) | 69.0 (22, 94) | 57.5 (22, 90) | 74.0 (24, 94) | 73.0 (18, 99) | 72.0 (18, 99) |
| **Female, n (%)** | 12 (41.4) | 34 (39.1) | 41 (36.0) | 20 (40.0) | 43 (41.3) | 175 (55.6) | 290 (50.3) |
| **Race, n (%)** |  |  |  |  |  |  |  |
| White | 24 (82.8) | 78 (87.9) | 96 (85.0) | 44 (88.0) | 78 (75.7) | 265 (84.7) | 484 (84.5) |
| **Diarrhoea reported ≤3 months preceding first fidaxomicin dose, n (%)** | | | | | | | |
| n | 27 | 82 | 108 | 48 | 100 | 303 | 554 |
| Yes | 26 (96.3) | 75 (91.5) | 100 (92.6) | 47 (97.9) | 85 (85.0) | 278 (91.7) | 507 (91.5) |
| **CDI** **episodes** **≤3 months preceding first fidaxomicin dose, n (%)** | | | | | | | |
| n | 29 | 88 | 119 | 51 | 109 | 319 | 590 |
| Yes | 5 (17.2) | 20 (22.7) | 21 (17.6) | 21 (41.2) | 17 (15.6) | 83 (26.0) | 141 (23.9) |
| **Treatment episodes per patient, n (%)** | | | | | | | |
| n | 29 | 87 | 114 | 50 | 104 | 315 | 576 |
| 1 | 29 (100.0) | 86 (98.9) | 110 (96.5) | 49 (98.0) | 100 (96.2) | 311 (98.7) | 564 (97.9) |
| 2 | 0 | 1 (1.1) | 3 (2.6) | 1 (2.0) | 3 (2.9) | 4 (1.3) | 10 (1.7) |
| 3 | 0 | 0 | 1 (0.9) | 0 | 1 (1.0) | 0 | 2 (0.3) |
| **Prescriptions per treatment episode, n (%)** | | | | | | | |
| n | 29 | 88 | 119 | 51 | 109 | 319 | 590 |
| 1 | 29 (100) | 83 (94.3) | 112 (94.1) | 49 (96.1) | 105 (96.3) | 308 (96.6) | 569 (96.4) |
| 2 | 0 (0.0) | 5 (5.7) | 7 (5.9) | 2 (3.9) | 4 (3.7) | 11 (3.4) | 21 (3.6) |

As some patients presented with >1 MCSI, the sum of the number of patients with each MCSI is greater than the total number of patients. Statistics and percentages are based on the total number of treatment episodes with known data (excluding missing and unknown data). CDI, *Clostridium difficile* infection; CI, confidence interval; IBD, inflammatory bowel disease; ICU, intensive care unit; MCSI, medical condition of specific interest; n, number of patients with known data; PI, principal investigator; SS, scoring system

# Supplementary Table 5 CDI history in the 3 months preceding the most recent fidaxomicin treatment episode

|  | **IBD (N=29)** | **Fulminant CDI-PI (N=88)** | **Fulminant CDI-SS (N=119)** | **Moderate-to-severe hepatic impairment (N=51)** | **Severe renal impairment (N=109)** | **No MCSI (N=319)** | **Total (N=590)^a^** |
| --- | --- | --- | --- | --- | --- | --- | --- |
| **Prior CDI episode in preceding 3 months^a^, n (%)** |  |  |  |  |  |  |  |
| n | 29 | 88 | 119 | 51 | 109 | 319 | 590 |
| Yes | 5 (17.2) | 20 (22.7) | 21 (17.6) | 21 (41.2) | 17 (15.6) | 83 (26.0) | 141 (23.9) |
| **Duration of prior CDI episode (days)** |  |  |  |  |  |  |  |
| n | 2 | 20 | 19 | 18 | 16 | 78 | 130 |
| Median (min, max) | 3.5 (3, 4) | 7.0 (3, 67) | 8.0 (3, 45) | 6.0 (3, 16) | 6.0 (2, 42) | 10.0 (1, 75) | 9.0 (1, 75) |
| **CDI was objectively confirmed, n (%)** |  |  |  |  |  |  |  |
| n | 5 | 23 | 23 | 23 | 22 | 111 | 180 |
| Yes | 5 (100.0) | 21 (91.3) | 21 (91.3) | 22 (95.7) | 20 (90.9) | 109 (98.2) | 174 (96.7) |
| **If yes, CDI confirmation method**^b^**, n (%)** |  |  |  |  |  |  |  |
| n | 5 | 21 | 21 | 22 | 20 | 109 | 174 |
| PCR | 3 (60.0) | 9 (42.9) | 7 (33.3) | 9 (40.9) | 6 (30.0) | 29 (26.6) | 51 (29.3) |
| Toxin detection | 2 (40.0) | 14 (66.7) | 17 (81.0) | 15 (68.2) | 14 (70.0) | 84 (77.1) | 131 (75.3) |
| Culture | 1 (20.0) | 10 (47.6) | 8 (38.1) | 13 (59.1) | 6 (30.0) | 41 (37.6) | 68 (39.1) |
| Other | 1 (20.0) | 0 (0.0) | 2 (9.5) | 1 (4.5) | 2 (10.0) | 7 (6.4) | 12 (6.9) |

As some patients presented with >1 MCSI, the sum of the number of patients with each MCSI is greater than the total number of patients. In the event of a patient having more than one treatment episode with fidaxomicin, treatment episodes are considered distinct if separated by more than 30 days from last dose of the earlier treatment episode to the first dose of the subsequent treatment episode. Statistics and percentages are based on the total number of treatment episodes with known data (excluding missing and unknown data). CDI, *Clostridium difficile* infection; IBD, inflammatory bowel disease; PCR, polymerase chain reaction; MCSI, medical condition of specific interest; N, number of treatment episodes; n, number of observations with known data; PI, principal investigator; SS, scoring system

^a^Up to the first dose of fidaxomicin

^b^Multiple diagnostic methods were often used simultaneously

# Supplementary Table 6 ECG assessments by MCSI: a) during the study observation period and b) shift summary

**a)**

| **Assessment** | **IBD (N=29)** | **Fulminant CDI-PI (N=87)** | **Fulminant CDI-SS (N=114)** | **Moderate-to-severe hepatic impairment (N=50)** | **Severe renal impairment (N=104)** | **No MCSI (N=315)** | **Total (N=576)** |
| --- | --- | --- | --- | --- | --- | --- | --- |
| **Baseline, n (%)** |  |  |  |  |  |  |  |
| n | 2 | 9 | 24 | 10 | 20 | 36 | 75 |
| Normal | 0 | 4 (44.4) | 5 (20.8) | 3 (30.0) | 4 (20.0) | 10 (27.8) | 19 (25.3) |
| Abnormal – NCS | 1 (50.0) | 5 (55.6) | 11 (45.8) | 3 (30.0) | 11 (55.0) | 19 (52.8) | 39 (52.0) |
| Abnormal – CS | 1 (50.0) | 0 | 8 (33.3) | 4 (40.0) | 5 (25.0) | 7 (19.4) | 17 (22.7) |
| **End of treatment, n (%)** | |  |  |  |  |  |  |
| n | 1 | 10 | 16 | 3 | 13 | 13 | 39 |
| Normal | 1 (100.0) | 5 (50.0) | 7 (43.8) | 3 (100.0) | 4 (30.8) | 2 (15.4) | 12 (30.8) |
| Abnormal – NCS | 0 | 4 (40.0) | 8 (50.0) | 0 | 7 (53.8) | 6 (46.2) | 19 (48.7) |
| Abnormal – CS | 0 | 1 (10.0) | 1 (6.3) | 0 | 2 (15.4) | 5 (38.5) | 8 (20.5) |
| **End of observation period, n (%)** | |  |  |  |  |  |  |
| n | 2 | 6 | 9 | 6 | 5 | 5 | 23 |
| Normal | 1 (50.0) | 5 (83.3) | 6 (66.7) | 3 (50.0) | 2 (40.0) | 2 (40.0) | 10 (43.5) |
| Abnormal – NCS | 1 (50.0) | 1 (16.7) | 2 (22.2) | 2 (33.3) | 3 (60.0) | 3 (60.0) | 12 (52.2) |
| Abnormal – CS | 0 | 0 | 1 (11.1) | 1 (16.7) | 0 | 0 | 1 (4.3) |

As some patients presented with >1 MCSI, the sum of the number of patients with each MCSI is greater than the total number of patients. Percentages are based on the total number of patients with known data (excluding missing and unknown data). CDI, *Clostridium difficile* infection; CS, clinically significant; IBD, inflammatory bowel disease; MCSI, medical condition of specific interest; N, number of patients; n, number of patients with known data; NCS, not clinically significant; PI, principal investigator; SS, scoring system

**b)**

| **Shift (baseline to post-baseline)** | **IBD (N=29)** | **Fulminant CDI-PI (N=87)** | **Fulminant CDI-SS (N=114)** | **Moderate-to-severe hepatic impairment (N=50)** | **Severe renal impairment (N=104)** | **No MCSI (N=315)** | **Total (N=576)** |
| --- | --- | --- | --- | --- | --- | --- | --- |
| **At end of treatment, n (%)** | |  |  |  |  |  |  |
| Abnormal to normal^a^ | 1/1 (100.0) | 1/2 (50.0) | 1/3 (33.3) | 0/0 | 1/4 (25.0) | 0/2 | 1/7 (14.3) |
| Normal to abnormal^b^ | 0/0 | 0/3 | 0/3 | 0/2 | 1/2 (50.0) | 1/1 (100.0) | 2/6 (33.3) |
| **At end of observation period, n (%)** | |  |  |  |  |  |  |
| Abnormal to normal^a^ | 1/1 (100.0) | 1/1 (100.0) | 1/4 (25.0) | 0/2 | 1/2 (50.0) | 0/0 | 1/6 (16.7) |
| Normal to abnormal^b^ | 0/0 | 0/4 | 0/4 | 0/2 | 0/1 | 0/1 | 0/6 |

As some patients presented with >1 MCSI, the sum of the number of patients with each MCSI is greater than the total number of patients. Percentages are based on the total number of treatment episodes with known data (excluding missing and unknown data). CDI, *Clostridium difficile* infection; IBD, inflammatory bowel disease; MCSI, medical condition of specific interest; N, number of patients; n, number of patients with known data; PI, principal investigator; SS, scoring system

^a^The denominator for shift to normal is the number of treatment episodes that had a post-baseline value at the visit and did not have normal at baseline

^b^The denominator for shift to abnormal is the number of treatment episodes that had a post-baseline value at the visit and did not have abnormal at baseline

# Supplementary Table 7 Fidaxomicin dosing and exposure by MCSI

|  | **IBD (N=29)** | **Fulminant CDI-PI (N=88)** | **Fulminant CDI-SS (N=119)** | **Moderate-to-severe hepatic impairment (N=51)** | **Severe renal impairment (N=109)** | **No MCSI (N=319)** | **Total (N=590)** |
| --- | --- | --- | --- | --- | --- | --- | --- |
| **Duration of dose (days)** |  |  |  |  |  |  |  |
| n | 29 | 88 | 119 | 51 | 109 | 318 | 589 |
| Median (min, max) | 10.0 (2, 14) | 10.5 (2, 26) | 11.0 (2, 26) | 11.0 (1, 20) | 11.0 (2, 25) | 11.0 (1, 41) | 11.0 (1, 41) |
| **Total number of doses taken** |  |  |  |  |  |  |  |
| n | 29 | 88 | 119 | 51 | 109 | 318 | 589 |
| Median (min, max) | 20.0 (4, 28) | 21.0 (2, 52) | 22.0 (2, 52) | 22.0 (1, 40) | 22.0 (2, 50) | 22.0 (1, 82) | 22.0 (1, 82) |
| **Followed recommended dose schedule, n (%)** | | |  |  |  |  |  |
| n | 29 | 88 | 119 | 51 | 109 | 319 | 590 |
| Yes | 24 (82.8) | 58 (65.9) | 79 (66.4) | 38 (74.5) | 70 (64.2) | 238 (74.6) | 431 (73.1) |
| **Completed regimen, n (%)** |  |  |  |  |  |  |  |
| n | 29 | 88 | 118 | 51 | 109 | 317 | 587 |
| Yes | 25 (86.2) | 63 (71.6) | 86 (72.9) | 39 (76.5) | 74 (67.9) | 252 (79.5) | 457 (77.9) |
| **Primary reason for discontinuation, n (%)** | | |  |  |  |  |  |
| **n** | 29 | 88 | 118 | 51 | 109 | 317 | 587 |
| Adverse event | 0 | 0 | 1 (0.8) | 0 | 1 (0.9) | 3 (0.9) | 4 (0.7) |
| Efficacy reason | 1 (3.4) | 4 (4.5) | 2 (1.7) | 0 | 2 (1.8) | 1 (0.3) | 7 (1.2) |
| Investigator decision | 0 | 10 (11.4) | 12 (10.2) | 5 (9.8) | 11 (10.1) | 22 (6.9) | 43 (7.3) |
| Death | 2 (6.9) | 9 (10.2) | 11 (9.3) | 3 (5.9) | 10 (9.2) | 7 (2.2) | 26 (4.4) |
| Other | 1 (3.4) | 4 (4.5) | 7 (5.9) | 5 (9.8) | 11 (10.1) | 40 (12.6) | 60 (10.2) |

As some patients presented with >1 MCSI, the sum of the number of patients with each MCSI is greater than the total number of patients. Statistics and percentages are based on the total number of treatment episodes with known data (excluding missing and unknown data). CDI, *Clostridium difficile* infection; IBD, inflammatory bowel disease; MCSI, medical condition of specific interest; N, number of treatment episodes; n, number of observations with known data; PI, principal investigator; SS, scoring system
